# Catheter-related candidemia and identification of causative *Candida* species in patients with cardiovascular disorder

**DOI:** 10.18502/cmm.4.2.63

**Published:** 2018-06

**Authors:** Shirinsadat Hashemi Fesharaki, Seyed Reza Aghili, Tahereh Shokohi, Mohammad Ali Boroumand

**Affiliations:** 1 Student Research Committee, School of Medicine, Mazandaran University of Medical Sciences, Sari, Iran; 2 Department of Medical Mycology, School of Medicine, Mazandaran University of Medical Sciences, Sari, Iran; 3 Invasive Fungi Research Center, Mazandaran University of Medical Sciences, Sari, Iran; 4 Research Committee of Pathology, School of Medicine, Tehran University of Medical Sciences, Tehran, Iran; 5 Cardiovascular Research Department, Tehran Heart Center, Tehran University of Medical Sciences, Tehran, Iran

**Keywords:** Candidiasis, Catheter-related candidemia, Nosocomial infection

## Abstract

**Background and Purpose:**

Catheter-related blood circulation infection is the most dangerous and serious side-effects of vascular catheters, which leads to the enhancement of the costs, mortality, and hospital stay duration, especially in the Intensive Care Unit. Regarding this, the aim of the current study was to identify the prevalence of catheter-induced candidemia in the Tehran Heart Center, a heart hospital in Tehran, Iran.

**Materials and Methods:**

This study was conducted on patients admitted to Tehran Heart Center for a minimum of 7 days during 18 months. To detect the fungal elements, blood culture and catheter culture were performed in the patients receiving central or peripheral venous catheter. Then, the polymerase chain reaction (PCR) was applied to determine the possible diagnosis.

**Results:**

The investigation of 223 samples led to the identification of a total of 15 (6.7%) yeast isolates obtained from 9 (60%), 4 (26.6 %), and 2 (13.4%) catheter, blood, and skin (of the catheter insertion areas) cultures, respectively. Out of nine *Candida* isolates obtained from the catheter samples, 1 (11.1%), 1 (11.1%), 2 (22.2%), and 5 (55.6%) cases were identified as *C. tropicalis,* *C. membranifaciens,* *C. glabrata*, and *C. albicans*, respectively, using the internal transcribed spacer region sequencing. Furthermore, the four yeasts isolated from the blood culture included *C. tropicalis*, *C. carpophila*, *C. membranifaciens, *and *Cryptococcus albidus*. Additionally, one case of *C. glabrata* and one case of *C. albicans* were isolated from the skin culture of the catheter insertion areas in patients with positive catheter culture. We reported two cases of catheter-related candidemia caused by *C. membranifaciens* and *C. tropicalis* on the basis of the genetic similarity of the species isolated from the blood and catheter. These cases were treated successfully with intravenous fluconazole and catheter removal.

**Conclusion:**

There is some evidence indicating the growing prevalence of non*-albicans Candida* infections. Many risk factors, including prior antibiotic therapy, use of a central venous catheter, surgery, and parenteral nutrition, are considered to be associated with candidemia in hospitalized heart failure patients. The identification of the route of infection in candidemia is difficult. In the current study, the positive blood and catheter cultures for *Candida* isolates and the similarity of the ITS region of ribosomal DNA sequence of *Candida* isolated from two patients confirmed the diagnosis of intravenous catheter-related candidemia.

## Introduction

During the past two decades, the incidence of nosocomial fungal infections has increased due to the use of wider and newer medical devices and technologies in developing countries, as well as in Iran. Heart failure disease and candidiasis have been shown to sometimes go hand in hand. Risk factors for invasive candidiasis in patients with heart disease may include prolonged stay in Intensive Care Units (ICUs), use of central venous catheters (CVC), treatment with broad-spectrum antibiotics and glucocorticoids, parenteral nutrition, and severe surgeries (e.g., cardiac valve repair or replacement) (1-3).

Venous catheters, despite their benefit applications, can predispose the patients to the colonization of fungal agents and lead to local infections, venous inflammations, or spread of infections in rare cases (4, 5). Invasive candidiasis is one of the most significant causes of mortality in hospitalized patients (6, 7). Furthermore, *Candida* is the fourth cause of nosocomial blood infection in the United States (8). 

Patients admitted to the special units of hospital, such as ICUs and Coronary Care Units (CCUs), are at high risk of Candida infections. The enhancement of candidemia incidence is accompanied with the increase in the size of general population (8, 9). Although *Candida albicans* is already the most prevalent fungal pathogenic species (10), there is an increase in the incidence frequency of non-*albicans* species, such as *C. tropicalis*, *C. guillermondii*, *C. glabrata,* and *C. parapsilosis,* as reported in many countries, especially among patients with immunosuppressive disorders (11-16). 

Some studies have shown that many non*-albicans Candida* species are usually resistant to common anti-fungal drugs, such as azoles (17-19). Patients with heart disorders admitted to hospitals receive catheters, most commonly CVCs. Catheters are commonly indicated as easy transmission routes for pathogens, including bacteria and fungi, such as *Candida *species (20, 21). 

The signs and symptoms of *Candida* infection depend upon the site of infection. However, if patients have candidemia, they may have fever, chills, skin rash, low blood pressure, headaches, neurological deficits, and abdominal pain (22). Additionally, catheter-induced candidemia may be associated with severe diseases, like infections, thrombosis, endocarditis, and meningitis (23). Positive results in blood culture or some new direct examinations (e.g., T_2_ *Candida* panel) indicate candidemia and invasive fungal infection.

As candidemia can cause a serious life-threating illness, treatment is usually begun when an infection is suspected. Candidemia treatment includes the detection of the source of infection, such as catheters, and if possible, removal of CVC and initiation of therapeutic course with medications. The medicines usually prescribed for this infection include azoles (e.g., fluconazole or voriconazole), amphotericin B, and echinocandin group (e.g., anidulafungin or caspofungin).

The type of administered drug depends on the severity of the patient’s illness and kind of *Candida* species causing the infection. Despite all these measures, candidemia accounts for a mortality rate of 40% (24). With this background in mind, the present study was conducted to identify the prevalence of catheter-related candidemia and its variant species in the ICUs of Tehran Heart Center, a heart hospital in Tehran, Iran. 

## Materials and Methods

This cross-sectional study was conducted on the patients referring to Tehran Heart Center during a period of 18 months (i.e., June 2010 to December 2011). All of the hospitalized patients suffering from cardiovascular complications with at least 7 days of hospital stay, intravenous indwelling catheter, and post-catheterization fever were included in the study. 

The collected data included the patients’ demographic information, past history, clinical and laboratory risk factors, intravenous nutrition, parenteral antibiotic, other parenteral drugs, presence of neutropenia (neutrophils<500 cells/ml), and type and frequency of catheters used during hospitalization, which were recorded in a checklist. The exclusion criteria were: 1) less than 7 days of hospital stay, 2) more than 7 days of hospital stay without any fever, and 3) disagreement to participate in the study. The study was approved by the Medical Research Ethics Committee of Mazandaran University of Medical Sciences, Mazandaran, Iran (ethical No. 91.8.17).

***Catheter tube culture for mycological studies***

Aseptically, the removed catheter was placed in a sterile tube containing normal saline, and then transferred to the laboratory. The external surface of the removed catheter was rolled on Brian Hart Infusion agar (Merck, Industrial Chemicals, NY, USA) containing 100 mg/L chloramphenicol (BHIC). To determine the intraluminal colonization of the catheters, they were shaken on sterile saline at the desired volume on a vortex for 3 min until the eventual colonization of the catheter tube was washed and separated. 

Subsequently, the suspension was centrifuged at 1,500 rpm for 5 min. The supernatant was removed without disturbing the cell button, and 1 ml sterile saline was added to the tube. The sediment suspension was inoculated into BHIC medium in two series and incubated for 7 days at 25ºC and 37ºC. The differential diagnosis of isolated yeast was performed by mycological procedures in the Invasive Fungi Research Center laboratories, Sari, Iran.

***Mycological studies of patients’ blood and skin samples***

Using an aseptic technique, 5 ml of blood was inoculated into 50 ml biphasic BHIC agar/broth (Kusha Faravar Giti, Karaj, Iran), and then vertically incubated at 37°C in aerobic conditions for 10 days. The bottles were upside down for inoculation with BHIC agar, twice on the first 2 days and once a day on the next days and checked for growth as previously described (25, 26). 

In addition, a smear was obtained from the liquid phase of the medium and stained by Gram staining. More than two blood samples were obtained over a day from each patient. A positive result was defined as the growth of the white colonies of yeast on the semisolid phase agar surfaces. For more accurate detection, at the end of the 10 days of incubation, 1 ml of the liquid phase was inoculated into two Sabouraud Dextrose agar plates (Quelab, Montreal, Canada) containing 100 mg/L chloramphenicol (SDAC). 

One of the plates was incubated at 25°C and the other one at 37°C. The growth of the yeast colonies in these plates were defined as positive. For the positive-detected cases, the results were confirmed by repeating sampling and blood cultures. Candidemia was confirmed if at least one blood culture was positive for *Candida *species in patients with compatible clinical signs and symptoms of infection. 

If the blood or catheter culture was positive, a sample was obtained from the skin of the catheter insertion area. The skin scraping samples were inoculated into two 10-cm diameter plates of SDAC, one of which was incubated at 25°C and the other one at 37°C. It should be noticed that the urine samples of the patients were investigated as well. 

***Identification of yeast species***

In the current study, the identification of yeast colony was accomplished by using the routine phenotypic methods, such as CHROMagar *Candida* (CHROMagar company, Paris, France), germ tube formation, morphological examination on a cornmeal agar (CMA, BBL Sparks, Maryland, USA) with Tween 80 (1%), and urease test. All yeast species were confirmed by DNA sequencing. DNA was extracted from the colony using glass beads and phenol-chloroform (27). 

The internal transcribed spacer (ITS) region of ribosomal DNA (rDNA) were amplified using two primers, namely ITS1 (5ʹ-TCCGTAGGTGAACCTGCGG-3ʹ) and ITS4 (5ʹ-TCCTCCGCTTATTGATATGC-3ʹ) (CinnaGen, Karaj, Iran). The sequencing results were evaluated using nucleotide basic local alignment search tool to determine the closest known relatives on the NCBI website (http://www.ncbi.nlm.nih.gov).

***Statistical Analysis***

The data were analyzed in SPSS software, version 19 (SPSS, Inc., Chicago, IL, USA). Mean and standard deviation were applied to describe the quantitative variables. The qualitative variables were reported as percentage and frequency. Chi-square test was used to determine the differences between the experimental factors and the association of the groups with each other. Additionally, non-parametric tests were used to compare the two groups. P-value less than 0.05 was considered statistically significant.

## Results

Out of 25,580 patients admitted to the hospital during our research period, a total of 223 patients were enrolled in the study based on the inclusion criteria. The mean age of the patients was 57.1±12.0 years (age range: 20-81 years). The incidence of heart failure was more common in the women and men aged 56-65 and 46-55 years, respectively. None of the patients had malignancy. Regarding the place of residence, all patients lived in town. 

According to the results, the frequency and duration of catheterization showed a direct relationship with yeast catheter colonization and incidence of primary candidemia (*P<0.05*). The duration of hospital stay, diabetes, and renal problems were the most frequent risk factors for candidemia in these patients. Table 1 presents the frequency of some characteristics of 223 patients.

Out of the 233 tested samples, a total of 15 (6.4%) yeast isolates were detected, 9 (60%), 4 (26.7%), and 2 (13.3%) cases of which were obtained from the catheter, blood, and skin sample cultures. The mean age of the patients with positive cultures was 56.7 years (age range: 22-74 years). Table 2 tabulates the results of phenotypic tests based on morphological and physiological characteristics and genotypic detection based on the sequence of the ITS region in 15 yeast positive cultures.

Out of the specimens, *Candida* species were the most frequent species obtained from CVC, blood, and skin samples. Based on ITS region sequencing as the gold standard identification method, the most common species identified in the 15 isolates from the catheter and skin samples included *C. albicans* (40%), followed by *C. glabrata* (20%). The other identified species were *C. membranifaciens* and *C. tropicalis *as separately obtained from both catheter and blood cultures of two patients. Additionally, *C. carpophila* and *Cryptococcus albidus* were isolated only from the blood cultures of two patients (Table 3)

Our results showed that *C. membranifaciens* isolated from both catheter and blood cultures of patient No. 93 had 100% similarity based on the ITS rDNA base pair. This similarity was also observed in *C. tropicalis* obtained from both catheter and blood cultures of patient No. 94. The quite similarity suggested that CVC can be the source of candidemia as called catheter-related candidemia. Accordingly, the results led to the detection of two cases of catheter-related candidemia caused by *C. membranifaciens* and *C. tropicalis* based on the genetic similarity of the species isolated from the blood and catheter samples. These cases were treated successfully with the administration of fluconazole and catheter removal.

**Table 1:** Characteristics of patients with catheter-related candidemia

**Characteristics**	**Age (year)** **mean±SD**	**Gender** **n (%)**		**Underlying disease** **n (%)**							
		**Male**	**Female**	**Diabetes**	**Renal failure**	**Pulmonary complication**	**Receiving intravenous antibiotics**	**>7 days of catheterization**	**Frequency of using catheter** **mean±SD**	**>7 days of hospitalization**
All patients	57.1±12.0	155 (69.5%)	68 (30.5%)	65 (29.1%)	9 (4.0%)	11 (4.9%)	153 (68.6)	39 (17.5%)	1.05±0.3	37 (16.6%)
*Candida *catheter colonization	59.6±12.0	5 (55.6%)	4 (44.4%)	4 (44.4)	0 (0.0%)	0 (0.0%)	8 (88. 9%)	5 (55.6%)	2.0 ± 13	4 (4.44%)
*P-*value	0.5	0.6	0.7	0.3	1.0	1.0	0.18	0.002	<0.0001	0.07
Primary candidemia	67.2±10.9	1 (25.0%)	3 (75%)	3 (75%)	1 (25%)	0 (0.0%)	2 (50%)	3 (75%)	2.2 ± 1.5	3 (75%)
*P*-value	0.09	0.14	0.08	0.04	0.04	1.0	0.4	0.002	<0.0001	0.007

**Table 2:** Origin and identity of isolates used in the comparative study

**Patients code**	**Source**	**Total ITS rDNA bp**	**Genotypic detection** **(Accession No.)**	**Germ tube Test**	**Urease test**	**Morphological characteristics of yeast on CMA**	**Colony color on CHROMagar Candida**	**Phenotypic detection**
25	Catheter	511	*Candida albicans* *MH746085*	+	N	Hyphae+ blastoconidia+ chlamydospores	Green	*Candida albicans*
143*	Blood	*598*	*Cryptococcus albidus* *MH734759*	N	+	Only blastoconidia	Pinkish white	*Candida *sp.
93*	Catheter	587	Candida membranifaciens* *MH734774*	N	+	Hyphae+ blastoconidia	Pinkish	*Candida *sp.
21*	Catheter	826	*Candida glabrata* *MH734758*	N	N	Only blastoconidia	White	*Candida *sp.
25*	Skin	503	*Candida albicans* *MH746019*	+	N	hyphae+ blastoconidia+ chlamydospores	Green	*Candida albicans*
62	Catheter	507	*Candida albicans* *MH746020*	+	N	hyphae+ blastoconidia+ chlamydospores	Green	*Candida albicans*
74	Catheter	498	*Candida albicans* *MH746086*	+	N	Hyphae+ blastoconidia+ chlamydospores	Green	*Candida albicans*
55	Blood	582	Candida carpophila* *MH746112*	N	N	Hyphae+ blastoconidia	Dark blue	*Candida. tropicalis*
94*	Blood	501	*Candida tropicalis* *MH744727*	N	N	Hyphae+ blastoconidia	Dark blue	*Candida tropicalis*
93*	Blood	594	Candida membranifaciens* *MH744723*	N	+	Hyphae+ blastoconidia	Pinkish	*Candida *Sp.
70	Catheter	517	*Candida albicans* *MH760814*	+	N	Hyphae+ blastoconidia	White to gray	*Candida *Sp.
9*	Skin	847	*Candida glabrata* *MH744728*	N	N	Only blastoconidia	White	*Candida *Sp.
9	Catheter	866	*Candida glabrata* *MH746080*	N	N	Only blastoconidia	White	*Candida *Sp.
94*	Catheter	522	*Candida tropicalis* *MH746021*	N	N	Hyphae+ blastoconidia	Blue	*Candida tropicalis*
140	Catheter	518	*Candida albicans* *MH773179*	+	N	Hyphae+ blastoconidia+ chlamydospores	Greenish	*Candida albicans*

* This obsolete species is a synonym of *C. guilliermondii, *+ =positive test, N= Negative test

**Table 3:** Frequency and percentage of yeast species isolated from patients based on the source of samples

**Sample**	**Yeast species**						
	***C. albicans***	***C. glabrata***	***C. tropicalis***	***C. membranifaciens***	***C. carpophila***	***Cryptococcus albidus***	**Total (%)**
Catheter	5	2	#1	1*	0	0	9 (60)
Blood	0	0	#1	1*	1	1	4 (26.6)
Skin	1	1	0	0	0	0	2 (13.4)
Total (%)	6 (40)	3 (20)	2 (13.3)	2 (13.3)	1 (6.7)	1 (6.7)	15 (100)

The patients with positive catheter culture results for yeast cells had the longest duration of ICU stay (*P=0.04*). Among the 9 patients with positive catheter culture results and 214 patients with negative catheter culture, 4 (44.4%) and 33 (15.4%) cases had the ICU stay of more than 15 days, respectively. Although the number of the yeast cells isolated from patients with coronary angioplasty were greater than that harvested from patients with heart valve disorders, this difference was not significant. 

Based on the hospital laboratory findings, the most common bacteria isolated from the catheter samples were *Staphylococcus epidermis* (42%) and *Staphylococcus aureus* (15%). Furthermore, the microbiological urine analysis led to the identification of yeast cells in only four samples, one case of which had positive catheter culture results for *C. albicans*. 

## Discussion

The prevalence of nosocomial candidemia has increased in recent decades. Accordingly, candidemia account for 10-20% of all nosocomial blood stream infections (10, 28, 29). The most important risk factors for candidemia are the presence of central vascular catheters, total parenteral nutrition, and antibacterial treatments (30, 31). Catheter-related infection is the most serious complication of central venous access and a leading cause of nosocomial infection in hospitalized heart failure patients (16, 32)

In the present study, there was no significant difference between the heart failure patients with positive yeast culture and those with negative yeast culture in terms of age and gender. However, the mean age of the positive yeast culture group was higher than that of the negative yeast culture one. According to the literature, hospitalized patients of higher age are prone to a higher risk of nosocomial fungal infection (33-35).

In our study, the statistically significant risk factors for the isolation of yeast from the blood or catheter samples included more than 7 days of catheterization, more than 7 days of hospitalization, and use of catheters for more than two times. This result is in line with those of other studies, indicating that the heart failure patients with catheters were more seriously affected by *Candida* species. Therefore, the health workers should take care of these patients more carefully (36, 37).

Our data also supported that diabetes was a major risk factor for heart failure patients (29.1%). In this regard, this disease was associated with a higher frequency of the *Candida* colonization of catheter and primary candidemia (44.4% and 75%, respectively). There is significant and consistent evidence in the literature indicating that diabetes as an important risk factor plays an important role in heart failure (38, 39) and invasive candidiasis (40, 41).

The detection rate of non*-albicans Candida* species, such as *C. glabrata*, *C. tropicalis*, *C. carpophila,* and *C. membranifacience*, excluding *C. albicans*, has gradually increased in the last two decades (42-45). In this study, *C. albicans* was the most frequently isolated species from all different samples. Nonetheless, non-*albicans* species (e.g., *C. membranifaciens* and *C. carpophila*) and *Cryptococcus albidus* were isolated from 75% of the positive blood culture samples. Therefore, the role of non-*albicans* *Candida* species and other yeast fungi should be considered in the detection of candidemia.

In the present study, yeast colonies were isolated from both catheter and blood samples of only two patients and showed similar molecular and genetic sequences; therefore, these two cases were identified as catheter-dependent candidemia. Recent advances in DNA gene sequencing techniques, not only have enabled us to eliminate the defects of the conventional methods, but also have provided a faster and more accurate diagnosis with high sensitivity and specificity (more than 90%) in many cases (46). 

Meanwhile, the barcoding and sequencing of various regions in the fungi are considered as reliable methods (47). Molecular diagnostic methods increase the ability to detect yeast organisms and determine the mutations, which are responsible for resistant to antifungal treatments (48). As a result, standardization and commercial molecular diagnostic techniques, which have a higher sensitivity, are necessary for the detection of yeast species, especially *Candida* species. 

Health care professionals should pay attention to the issue that long-term hospital stay is one of the risk factors for infection with yeast fungi, such as *Candida *species. Consequently, all patients should be screened for candidiasis at different stages of the hospital process. This is especially needed in the patients receiving a variety of catheters (e.g., intravenous, peritoneal, and urethral) or surgically treated and prescribed to use broad-spectrum antibiotics. Consistent with the results of many other studies (49-52), our findings showed that the role of *non-albicans* species of *Candida* in the incidence of candidemia in hospitalized patients is on a growing trend. 

## Conclusion

Catheter-induced candidemia has a good prognosis if it is timely diagnosed, and a proper and rapid anti-fungal treatment is applied, along with the removal of the catheter. However, this infection would lead to fungal overload and spread of organisms to regions distal to catheter, especially in patients with poor performance status, such as those with heart disorders, thereby increasing the possibility of infection in other body parts. The identification of a rapid, reliable, and accurate test can reduce the diagnostic time. This can improve the quality of care for hospitalized patients, and consequently reduce the cost of health care, morbidity, and mortality.
